# The 29.5 kb *APOBEC3B* Deletion Polymorphism Is Not Associated with Clinical Outcome of Breast Cancer

**DOI:** 10.1371/journal.pone.0161731

**Published:** 2016-08-23

**Authors:** Jingjing Liu, Anieta M. Sieuwerts, Maxime P. Look, Michelle van der Vlugt-Daane, Marion E. Meijer-van Gelder, John A. Foekens, Antoinette Hollestelle, John W. M. Martens

**Affiliations:** 1 Department of Medical Oncology, Erasmus MC Cancer Institute, Erasmus University Medical Center, Rotterdam, the Netherlands; 2 Cancer Genomics Netherlands, Utrecht, the Netherlands; Sudbury Regional Hospital, CANADA

## Abstract

Increased *APOBEC3B* mRNA levels are associated with a hypermutator phenotype and poor prognosis in ER-positive breast cancer patients. In addition, a 29.5 kb deletion polymorphism of *APOBEC3B*, resulting in an *APOBEC3A-B* hybrid transcript, has been associated with an increased breast cancer risk and the hypermutator phenotype. Here we evaluated whether the *APOBEC3B* deletion polymorphism also associates with clinical outcome of breast cancer. Copy number analysis was performed by quantitative PCR (qPCR) in primary tumors of 1,756 Dutch breast cancer patients. The *APOBEC3B* deletion was found in 187 patients of whom 16 carried a two-copy deletion and 171 carried a one-copy deletion. The prognostic value of the *APOBEC3B* deletion for the natural course of the disease was evaluated among 1,076 lymph-node negative (LNN) patients who did not receive adjuvant systemic treatment. No association was found between *APOBEC3B* copy number values and the length of metastasis-free survival (MFS; hazard ratio (HR) = 1.00, 95% confidence interval (CI) = 0.90–1.11, *P* = 0.96). Subgroup analysis by ER status also did not reveal an association between *APOBEC3B* copy number values and the length of MFS. The predictive value of the *APOBEC3B* deletion was assessed among 329 ER-positive breast cancer patients who received tamoxifen as the first-line therapy for recurrent disease and 226 breast cancer patients who received first-line chemotherapy for recurrent disease. No association between *APOBEC3B* copy number values and the overall response rate (ORR) to either tamoxifen (odds ratio (OR) = 0.88, 95% CI = 0.69–1.13, *P* = 0.31) or chemotherapy (OR = 0.97, 95% CI = 0.71–1.33, *P* = 0.87) was found. Thus, in contrast to *APOBEC3B* mRNA levels, the *APOBEC3B* deletion polymorphism has neither a prognostic nor a predictive value for breast cancer patients. Although a correlation exists between *APOBEC3B* copy number and mRNA expression, it is relatively weak. This suggests that other mechanisms exist that may affect and therefore determine the prognostic value of *APOBEC3B* mRNA levels.

## Introduction

Breast cancer, like most cancer types, is a heterogeneous disease. The heterogeneous nature of breast cancer, however, provides challenges for identifying appropriate markers for disease susceptibility and progression, as well as treatment selection. Accordingly, transcriptional profiling has identified five molecular subtypes of breast cancer, which differ in prognosis, efficacy of treatment and preferred site of metastasis [1–5]. More recently, the catalogues of mutations across human cancers have provided us insight into the mutational processes that drive tumorigenesis [6,7]. For breast cancer, five distinct mutational signatures have been defined that contribute in varying degree to the final mutational catalogue of a breast tumor [7]. One of the most pronounced mutational processes impacting breast tumorigenesis is driven by the AID/APOBEC family of cytidine deaminases and gives rise to C>T and C>G substitutions at TpCpN nucleotides. Moreover, this mutational process associates with regional somatic hypermutation or kataegis [6–8].

The *APOBEC3* gene cluster is located on chromosome 22q13.1-q13.2 and harbors seven *APOBEC3* genes that have evolved in primates (*i*.*e*. *APOBEC3A*, *APOBEC3B*, *APOBEC3C*, *APOBEC3D*, *APOBEC3F*, *APOBEC3G* and *APOBEC3H*) [9]. APOBEC3s play a role in intracellular defense through restriction of retroviral infections, but also of infections from the cancer-associated hepatitis B virus, the human papilloma virus and human T-lymphotropic virus [10]. Moreover, APOBEC3A, APOBEC3B, APOBEC3C and APOBEC3F are also able to inhibit LINE1 retrotransposition [11,12]. Besides its role in innate immunity, *APOBEC3B* has recently been identified as an endogenous source of mutation in breast cancer [13]. *APOBEC3B* mRNA expression was found to be upregulated in most breast cancers and tumors expressing high levels of *APOBEC3B* had a 2-fold increase in mutations compared with tumors expressing low *APOBEC3B* levels. This suggests that APOBEC3B, at least in part, underlies the APOBEC-driven mutational process in breast cancer, but also in other cancers [13,14]. In line with these findings, high levels of *APOBEC3B* mRNA were associated with a shorter disease-free survival in ER-positive, LNN, systemically untreated patients, as well as with earlier recurrence in luminal subtype patients and with a more aggressive phenotype in Japanese breast cancers [15–17]. Moreover, *APOBEC3B* expression has been reported to be associated with a strong enrichment of mitotic and cell cycle-related genes [16].

A 29.5 kb germline deletion between the fifth exon of *APOBEC3A* and the eighth exon of *APOBEC3B* has been identified that essentially removes the complete *APOBEC3B* coding region from the genome and generates a fusion transcript of *APOBEC3A* with the 3’untranslated region (UTR) of *APOBEC3B* [18]. With a worldwide frequency of 22.5%, the frequency of the germline *APOBEC3B* deletion variant varies widely among the different ethnic groups, ranging from being rare in African and European populations (i.e. 0.9% and 6%, respectively) to being common in Asian and American populations (i.e. 36.9% and 57.7%, respectively) [18]. Through a genome-wide association study of copy number variation, Long *et al*. found that the *APOBEC3B* deletion variant was associated with an increased risk to develop breast cancer in Chinese women [19]. This finding was replicated among European [20] and Southeast Iranian women [21], but not among Swedish women [22]. Interestingly, carriers of the *APOBEC3B* deletion were shown to have a greater *APOBEC3A* mRNA stability resulting in higher APOBEC3A levels, increased activity of APOBEC-driven mutational processes and more severe DNA damage [23,24]. However, the *APOBEC3B* deletion polymorphism was not associated with the survival of breast cancer patients [16,22]. Thus, despite that *APOBEC3B* overexpression and the *APOBEC3B* deletion variant both result in a hypermutation phenotype, there seems to be a difference between the two mechanisms and their association with clinical outcome.

To investigate the relation between the *APOBEC3B* deletion polymorphism and clinical outcome of 1,756 Dutch breast cancer patients, we explored four different clinical cohorts: LNN patients who did not receive any adjuvant treatment, lymph node positive (LNP) patients who did receive adjuvant systemic treatment, hormone-naive ER-positive patients who received tamoxifen as first-line therapy for recurrent disease and patients that received first-line chemotherapy for recurrent disease. Furthermore, to investigate the relation between the clinical outcomes based on the *APOBEC3B* deletion polymorphism versus *APOBEC3B* overexpression, we investigated the correlation between *APOBEC3B* copy number and *APOBEC3B* mRNA expression.

## Materials and Methods

### Study population

The retrospective study cohort consisted of 1,756 breast cancer patients who underwent surgery for an invasive primary breast cancer between 1978 and 2001. Inclusion criteria were no neoadjuvant treatment, no experience of a previous other cancer (except for basal cell carcinoma or stage Ia/Ib cervical cancer), a minimum of 100 mg of freshly frozen primary tumor tissue available for downstream DNA isolation and DNA available from tissue with a tumor cell nuclei percentage ≥ 30%. Cytosolic ER and PR levels were determined by ligand binding assay or enzyme immunoassay [25,26]. ER and/or PR positivity was defined by ≥10 fmol/mg cytosolic protein and *ERBB2* overexpression was defined by a reverse transcriptase qPCR expression level ≥18 [27]. In total, 796 patients underwent breast-conserving lumpectomy and 960 patients underwent modified radical mastectomy. In addition, 215 patients received adjuvant hormonal therapy, 308 patients received adjuvant chemotherapy and 6 patients received both adjuvant hormonal therapy and chemotherapy. There were 1,713 M0 patients and 43 M1 patients. The median age at the time of the primary surgery was 54 years, while the median age at the start of first-line treatment was 55 years. The clinicopathological variables of the patients are shown in Table 1.

**Table 1:** Association of *APOBEC3B* copy number status with clinicopathological variables in 1,756 primary breast cancers.

Variables	Deleted		Balanced		Amplified		*P*-value
Total number	187		1260		309		
Age (in years)							0.54
≤ 40	23	(12.3%)	160	(12.7%)	48	(15.5%)	
41–55	82	(43.9%)	498	(39.5%)	116	(37.5%)	
56–70	62	(33.2%)	412	(32.7%)	99	(32.0%)	
>70	20	(10.7%)	190	(15.1%)	46	(14.9%)	
Menopausal status							0.31
Premenopausal	89	(47.6%)	554	(44.0%)	149	(48.2%)	
Postmenopausal	98	(52.4%)	706	(56.0%)	160	(51.8%)	
Tumor size							1.00
pT1	71	(38.0%)	469	(37.2%)	115	(37.2%)	
pT2 + Unknown	96	(51.3%)	661	(52.5%)	163	(52.8%)	
pT3 + pT4	20	(10.7%)	130	(10.3%)	31	(10.0%)	
Nodal status							0.041
N0	117	(63.6%)	793	(63.5%)	173	(56.5%)	
N1-3	23	(12.5%)	212	(17.0%)	67	(21.9%)	
N>3	44	(23.9%)	244	(19.5%)	66	(21.6%)	
Tumor grade							0.052
Good/Moderate	34	(23.8%)	193	(21.9%)	66	(29.6%)	
Poor	109	(76.2%)	689	(78.1%)	157	(70.4%)	
Tumor histology							0.29
IDC	129	(83.2%)	833	(80.6%)	196	(78.4%)	
ILC	10	(6.5%)	119	(11.5%)	30	(12.0%)	
Other	16	(10.3%)	82	(7.9%)	24	(9.6%)	
ER status							0.24
Positive	130	(70.3%)	951	(75.8%)	226	(73.9%)	
Negative	55	(29.7%)	303	(24.2%)	80	(26.1%)	
PR status							0.99
Positive	114	(65.5%)	779	(65.8%)	187	(65.6%)	
Negative	60	(34.5%)	404	(34.2%)	98	(34.4%)	
*HER2* status							0.59
Positive	18	(12.6%)	150	(15.7%)	39	(16.2%)	
Negative	125	(87.4%)	807	(84.3%)	201	(83.8%)	
Adjuvant systemic therapy							0.17
None	133	(73.5%)	856	(69.9%)	191	(63.0%)	
Chemotherapy	26	(14.4%)	219	(17.9%)	63	(20.8%)	
Hormonal therapy	22	(12.2%)	146	(11.9%)	47	(15.5%)	
Both	0	(0%)	4	(0.3%)	2	(0.7%)	

Note: For nodal status, tumor grade, tumor histology, ER, PR and *HER2* status and adjuvant systemic therapy the number of patients do not add up to 1,756 because there were missing values for these variables. In addition, for the adjuvant systemic treatment variable: some patients were not eligible for adjuvant treatment because they were had distant metastasis at the time of primary tumor diagnosis.

**Fig 1:**
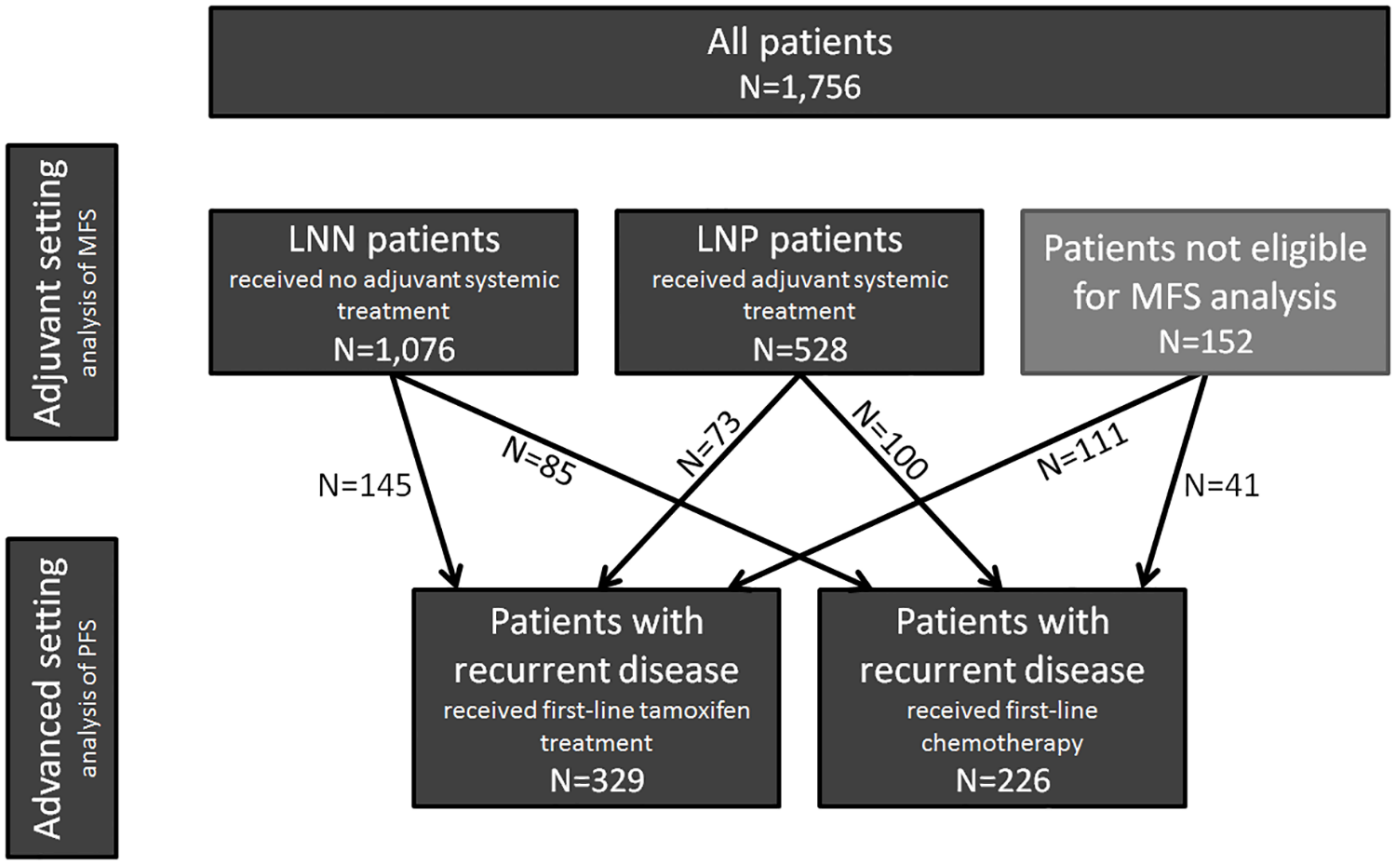
Schematic overview of study cohort. In total, this retrospective study consists of 1,756 primary breast cancers from patients who underwent surgery between 1978 and 2001. Inclusion criteria are specified in the Materials and Methods section. In the adjuvant setting, there were 1,076 lymph node negative (LNN) patients who did not receive adjuvant systemic treatment and 528 lymph node positive (LNP) patients who received adjuvant systemic treatment for the analysis of MFS. In the advanced setting, a group of 329 hormone-naive patients with ER-positive breast cancer received first-line tamoxifen for recurrent disease. Of these, 145 patients came from the LNN patients group and 73 came from the LNP patients group. The remaining 111 patients in this group did not qualify for MFS analysis (*i*.*e*. 82 patients did not fulfill LNN or LNP study eligibility criteria and 29 patients already presented with metastasis at the time of diagnosis). Furthermore, a group of 226 patients received first-line chemotherapy for recurrent disease. Of these, 85 patients came from the LNN patients group and 100 came from the LNP patients group. The remaining 41 patients in this group did not qualify for MFS analysis (*i*.*e*. 27 patients did not fulfill LNN or LNP study eligibility criteria and 14 patients already presented with metastasis at the time of diagnosis).

The total study cohort consists of four specific studies that were grouped together: 1) 1,076 LNN patients who did not receive any adjuvant systemic treatment, 2) 528 LNP patients who received adjuvant systemic treatment, 3) 329 hormone-naive ER-positive patients who received first-line tamoxifen therapy for recurrent disease and 4) 226 patients who received first-line chemotherapy for recurrent disease as detailed in Fig 1. We included all eligible patients fulfilling the study criteria and the general inclusion criteria specified above. As a consequence 403 patients were included in two studies (*i*.*e*. one study in the adjuvant setting and one study in the advanced setting). The total study population, however, cannot be considered a pure consecutive series, since systemically treated LNN patients were not included. The reason for this is that we especially wished to study the association of *APOBEC3B* copy number with the natural course of the disease in LNN patients, not potentially confounded by adjuvant systemic therapy.

Routine postsurgical follow-up and the definition of time to metastasis for LNN and LNP patients were as described previously [28]. The median follow-up for the 1,604 LNN and LNP patients included in the prognostic studies for the analysis of metastasis-free survival (MFS) was 114 months (range 10–354 months). Of these 1,604 patients, 686 (42.8%) had developed a distant metastasis and 691 (43.1%) of the 1,604 patients died during follow up. More specifically, 602 (37.5%) patients died after disease recurrence, whereas 89 (5.5%) patients died without evidence of disease recurrence at last follow-up. These 89 patients were censored at the date of last follow-up in the analysis of MFS.

Criteria for follow up and response to tamoxifen therapy were defined by standard International Union Against Cancer (Geneva, Switzerland) criteria of tumor response [29]. Complete and partial remission (together objective response) was observed in 11 and 48 patients, respectively, whereas 79 patients had progressive disease. From the patients with stable disease, 171 had no change for > 6 months, whereas 20 patients had no change for ≤ 6 months. According to the advice of the European Organization for Research and Treatment of Cancer [30], we defined overall response as complete and partial remission including stable disease > 6 months. As a result, 230 patients were classified as responders to tamoxifen and 99 patients showed no response to tamoxifen. The median follow up of patients after start of tamoxifen therapy was 49 months (range: 4–176 months). At the end of the follow up, 304 (92.4%) patients had developed tumor progression and were counted as events in the analysis of progression-free survival (PFS) and 264 patients had died.

Criteria for follow up and response to chemotherapy were similar to those for tamoxifen therapy with the exception that not all patients were chemotherapy-naïve. In fact, 45 out of 226 patients had received adjuvant chemotherapy. Of those, 33 patients received cyclophosphamide/methotrexate/5-fluorouracil (CMF), 11 patients received anthracycline-based chemotherapy and 1 patient received both. Complete and partial remission was observed in 12 and 69 patients, respectively, whereas 66 patients had progressive disease. From the 77 patients with stable disease, 54 had no change for > 6 courses, whereas 23 patients had no change for ≤ 6 courses. We defined overall response as complete and partial remission including stable disease >6 courses. As a result, 135 patients were classified as responders to chemotherapy and 89 patients showed no response to chemotherapy. For two patients the type of response was ambiguous. The median follow up of patients after start of chemotherapy was 30 months (range: 4–153 months). Fifty-two patients received consolidation therapy after chemotherapy and PFS was right censored at two months after the start of consolidation therapy. At this time point, 138 patients had developed tumor progression and were counted as events in the analysis of PFS. At the end of follow up, 210 patients had died.

This study was approved by the Medical Ethical Committee of the Erasmus Medical Center Rotterdam, the Netherlands (MEC 02.953). As this is a retrospective study using remaining material from surgical resection of the patient’s primary tumor, obtaining informed consent from the patient was not required provided patient records were anonymized and de-identified prior to analysis. Herewith, we adhered to the Code of Conduct of the Federation of Medical Scientific Societies in the Netherlands (http://www.fmwv.nl). Results are reported in accordance with the REMARK criteria on clinical reporting [31].

### Copy number analysis

Copy number analysis for *APOBEC3B* was performed on genomic DNA isolated from fresh-frozen primary tumor sections by qPCR on a Mx3000/3005P machine (Agilent Technologies, Santa Clara, CA). Briefly, genomic DNA was isolated from two to ten 30 μm cryostat sections (5–20 mg) with the NucleoSpin Tissue kit (Macherey–Nagel, Düren, Germany) according the protocol provided by the manufacturer. DNA quantity and quality was assessed by Nanodrop and the Quant-iT PicoGreen dsDNA HS Assay Kit (Thermo Scientific, Waltham, MA). Next, 0.5X Taqman Copy Number Assay for the *APOBEC3B* gene (Hs04504055_cn; Thermo Scientific), 0.5X Taqman Copy Number Reference Assay (*i*.*e*. for the *RNase P* gene; Thermo Scientific) and 0.5X ABsolute qPCR Mix, low ROX (Thermo Scientific) were added to 20 ng of genomic DNA in a final volume of 17 μl. Cycling conditions were: 1 cycle of 15 minutes at 95°C and 45 cycles of 15 seconds at 92°C and 1 minute at 60°C. The MxPro qPCR software v4.10 (Agilent) was used to calculate the cycle threshold (Ct) values. Relative quantification analysis was performed within the CopyCaller v2.0 software (Thermo Scientific). For this, the ΔCt value was calculated for each sample by subtracting the Ct value for the target gene (*i*.*e*. *APOBEC3B*) from the Ct value of the reference gene (*i*.*e*. *RNase P*). In the case of no Ct value for *APOBEC3B* after 45 cycles, while the *RNase P* gene was successfully amplified within 32 cycles for that sample, the ΔCt value could not be quantified and the sample was designated to have a two-copy deletion of the *APOBEC3B* gene. For samples where the ΔCt was quantified, ΔCt values were converted to calculated copy number values by the CopyCaller software as detailed in S1 Fig. Then, samples with calculated copy numbers ≤ 0.2 were called as two-copy deletion of the *APOBEC3B* gene, samples with calculated copy numbers >0.20 and ≤ 1.41 as one-copy deletion of the *APOBEC3B* gene, samples with calculated copy numbers > 1.41 and ≤ 3.44 as no copy number change or balanced and samples with calculated copy numbers > 3.44 as amplified for the *APOBEC3B* gene. Furthermore, 325 samples were measured in duplicate distributed over the 96-well sample plates and each 96-well plate included genomic DNA from breast cancer cell line OCUB-F as a control since this cell line has a two-copy deletion of *APOBEC3B*.

### Expression analysis

*APOBEC3B* mRNA expression analysis has been performed previously for 1,491 breast cancer patients [15]. Extraction of RNA, synthesis of cDNA, reverse transcriptase quantitative PCR and quantification of transcripts was also described before [32]. Out of the 1,756 patients for whom we performed *APOBEC3B* copy number analysis in the current study, *APOBEC3B* mRNA expression data was available for 1,132 patients.

### Statistical analyses

Because the number of patients carrying a two-copy deletion of *APOBEC3B* was small (N = 16, 0.91%), we grouped patients with one-copy and two-copy deletions together. A χ^2^ or Fisher’s exact test (when the expected frequency was ≤ 5 in any of the groups) was used to evaluate the association between the *APOBEC3B* copy number status (*i*.*e*. deleted, balanced or amplified) and the clinicopathological variables. To assess the association between the *APOBEC3B* copy number status and the ORR to either first-line tamoxifen treatment or first-line chemotherapy, we used a logistic regression model to calculate ORs and their 95% CIs. For visualization purposes, we performed survival analysis by the Kaplan-Meier method. The difference between survival curves for patients with either a deletion, no copy number change, or an amplification was calculated using the 3-sample logrank test. In addition, univariate Cox proportional hazards regression models including continuous calculated copy number values as covariate were performed to assess the association between *APOBEC3B* copy number and survival times (*i*.*e*. MFS in the adjuvant setting and PFS in the advanced setting). Finally, the correlation between *APOBEC3B* copy number and *APOBEC3B* expression was evaluated using Spearman’s rank correlation. All *P*-values were two-sided and *P*-values < 0.05 were considered to be statistically significant. Analyses were performed using R, version 3.2.3, except for the power and sample size calculations shown in the Discussion. For these, we used the stpower cox tool in Stata version 13.1 (StataCorp, College Station, TX) assuming an alpha of 0.05, a beta of 0.2 (*i*.*e*. only in sample size calculations) and similar covariate standard deviations, allele frequencies, event probabilities and sample sizes (*i*.*e*. only in power calculations) as observed in the current study.

## Results

We have performed *APOBEC3B* copy number analyses among 1,756 primary breast cancers and found no copy number change among 1,260 breast cancers. A two-copy deletion was identified in 16 (0.91%) breast cancers, whereas 171 (9.74%) breast cancers had a one-copy deletion. In addition, we detected an amplified *APOBEC3B* gene locus in 309 (17.26%) breast cancers. The minor allele frequency of the *APOBEC3B* deletion was 5.8% (203/3512) which is similar to the expected frequency in European population (*i*.*e* 6.5%) [18].

Next, we evaluated the association between the *APOBEC3B* copy number status and the clinicopathological variables of the 1,756 breast cancer patients. We found that *APOBEC3B* copy number status was significantly associated with nodal status (*P* = 0.041), but not with age, menopausal status, tumor size, tumor grade, tumor histology, ER, PR and *HER2* status, and adjuvant systemic therapy (Table 1). Despite the significant association between *APOBEC3B* copy number status and nodal status, however, no meaningful trend was observed (Table 1).

**Fig 2:**
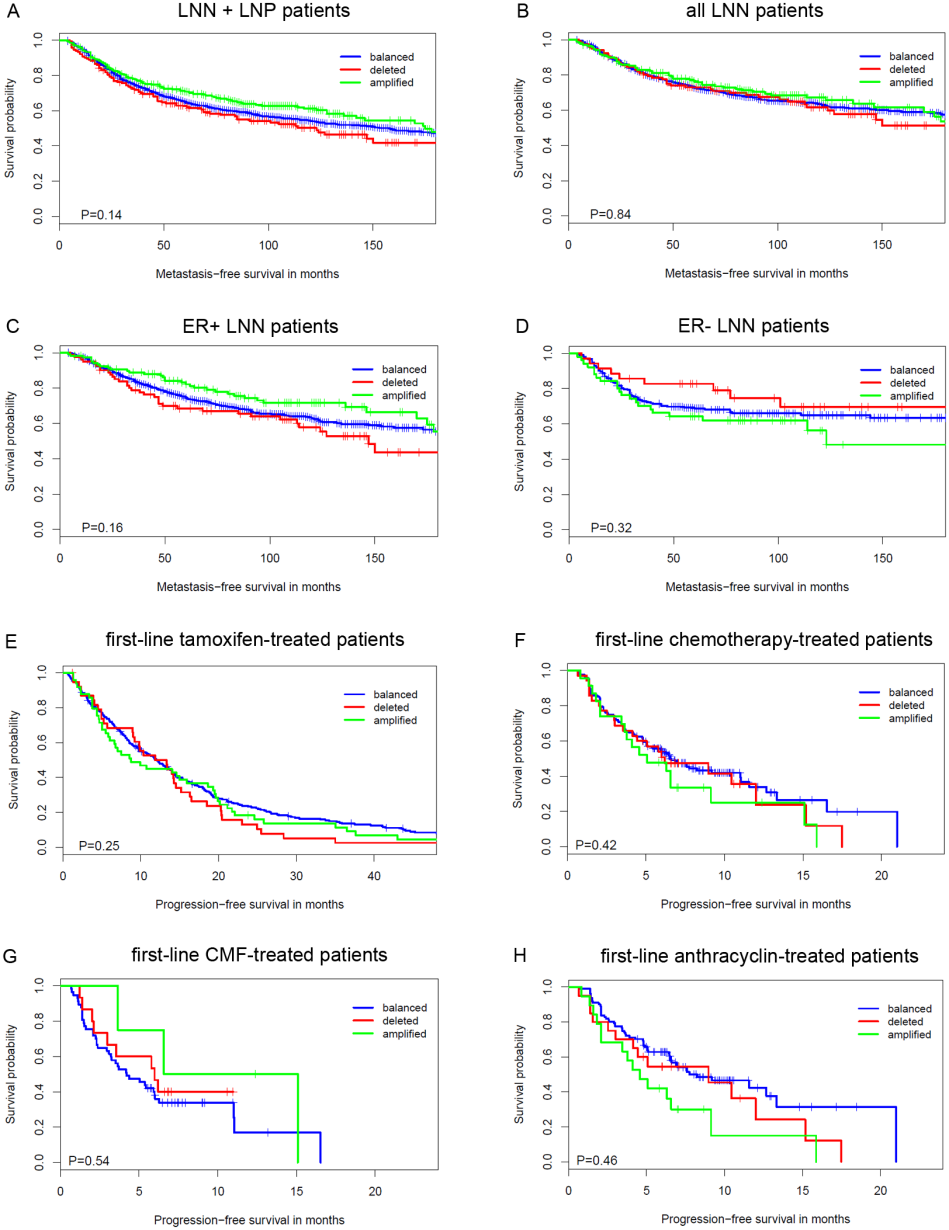
Kaplan-Meier survival analysis as a function of APOBEC3B copy number status. (A) In 1,604 patients of the lymph node negative (LNN) and lymph node positive (LNP) cohort combined. (B) In 1,076 LNN patients who did not receive any adjuvant systemic treatment. (C) In 769 ER-positive LNN patients who did not receive any adjuvant systemic treatment. (D) In 300 ER-negative LNN patients who did not receive any adjuvant systemic treatment. (E) In 329 ER-positive breast cancer patients who received first-line tamoxifen for recurrent disease. (F) In 226 breast cancer patients who received first-line chemotherapy for recurrent disease. (G) In 76 breast cancer patients who received first-line CMF-based chemotherapy for recurrent disease. (H) In 150 breast cancer patients who received first-line anthracycline based chemotherapy for recurrent disease. Differences between the survival curves were calculated with the 3-sample logrank test.

To assess whether *APOBEC3B* copy numbers associate with clinical outcome in the adjuvant setting, we performed Kaplan-Meier survival analysis and Cox regression analysis in 1,604 LNN and LNP patients. No association between *APOBEC3B* copy number status and the length of MFS was found (*P* = 0.14; Fig 2A). Moreover, calculated copy number values were also not associated with the length of MFS (HR = 0.95, 95% CI = 0.88–1.02, *P* = 0.17; Table 2). Because all LNP patients were treated with adjuvant systemic therapy and we specifically wanted to evaluate the prognostic value of *APOBEC3B* copy number, we repeated these analyses in the cohort of 1,076 LNN patients that had not received any adjuvant systemic treatment. Again, we found no association between *APOBEC3B* copy number status and the length of MFS (*P* = 0.84; Fig 2B), nor did we find an association between *APOBEC3B* calculated copy number values and the length of MFS (HR = 1.00, 95% CI = 0.90–1.11, *P* = 0.96; Table 2). Also when we performed subgroup analysis in 769 ER-positive or 300 ER-negative untreated LNN patients (*i*.*e*. ER status was not available for 7 patients), *APOBEC3B* copy numbers did not appear to have any prognostic value (Fig 2C and 2D, Table 2).

**Table 2:** Univariate Cox regression analysis to evaluate the association of calculated *APOBEC3B* copy number values with the length of MFS.

Study cohort	N Patients	N Events	Univariate analysis	
			HR (95% CI)	*P*-value
LNN+LNP				
All patients	1,604	686	0.95 (0.88–1.02)	0.17
LNN				
All patients	1,076	366	1.00 (0.90–1.11)	0.96
ER+ patients	769	263	0.93 (0.82–1.06)	0.29
ER- patients	300	101	1.13 (0.94–1.35)	0.20

N, number of; HR, hazard ratio; CI, confidence interval; LNN, lymph node negative; LNP, lymph node positive.

Note: ER status was not available for 7 out of 1,076 patients.

To evaluate the predictive value of *APOBEC3B* copy numbers, we had two cohorts of breast cancer patients available that were treated with first-line therapy for recurrent disease. First, we evaluated whether the calculated *APOBEC3B* copy number values could predict the clinical outcome of first-line tamoxifen therapy in a cohort of 329 hormone-naive breast cancer patients with ER-positive primary breast cancer. No significant association was observed between *APOBEC3B* copy number and the ORR for tamoxifen therapy (OR = 0.88, 95% CI = 0.69–1.13, *P* = 0.31; Table 3). Moreover, *APOBEC3B* copy number status was not associated with the length of PFS (*P* = 0.25; Fig 2E), nor were calculated *APOBEC3B* copy number values associated with the length of PFS (HR = 1.00, 95% CI = 0.88–1.14, *P* = 0.96; Table 4) in this cohort. Thus, *APOBEC3B* copy number is not a suitable biomarker to predict the type of response to tamoxifen therapy.

**Table 3:** Univariate logistic regression analysis of the overall response rate in patients treated with first-line tamoxifen and in patients treated with first-line chemotherapy for recurrent disease.

Study cohort	N Patients	Univariate analysis	
		OR (95% CI)	*P*-value
First-line tamoxifen			
All patients	329	0.88 (0.69–1.13)	0.31
First-line chemotherapy			
All patients	224	0.96 (0.71–1.31)	0.80
CMF-treated patients	75	1.25 (0.70–2.21)	0.45
Anthracyclin-treated patients	149	0.79 (0.53–1.16)	0.22

N, number of; OR, odds ratio; CI, confidence interval; CMF, cyclophosphamide/methotrexate/5-fluorouracil.

Note: the type of response was ambiguous for 2 patients

**Table 4:** Univariate Cox regression analysis to evaluate the association of calculated *APOBEC3B* copy number values with the length of PFS.

Study cohort	N Patients	N Events	Univariate analysis	
			HR (95% CI)	*P*-value
First-line tamoxifen				
All patients	329	304	1.00 (0.88–1.14)	0.96
First-line chemotherapy				
All patients	226	138	1.06 (0.88–1.29)	0.53
CMF-treated patients	76	52	0.91 (0.65–1.28)	0.58
Anthracyclin-treated patients	150	86	1.19 (0.93–1.52)	0.17

N, number of; HR, hazard ratio; CI, confidence interval; CMF, cyclophosphamide/methotrexate/5-fluorouracil.

Note: The length of PFS was censored at two months after the start of consolidation therapy.

Next, we evaluated whether calculated *APOBEC3B* copy number values could predict the outcome of first-line chemotherapy in a cohort of 226 breast cancer patients. The calculated *APOBEC3B* copy number values were, however, not found to be associated with the ORR for chemotherapy (OR = 0.96, 95% CI = 0.71–1.31, *P* = 0.80; Table 3). In addition, neither *APOBEC3B* copy number status, nor calculated *APOBEC3B* copy number values were associated with the length of PFS in these patients (*P* = 0.42; Fig 2F and HR = 1.06, 95% CI = 0.88–1.29, *P* = 0.53; Table 4, respectively). The lack of a significant association with the ORR to chemotherapy and the length of PFS was also observed when performing subgroup analysis by type of chemotherapy (*i*.*e*. CMF versus anthracyclines; Table 3, Fig 2G and 2H, Table 4). *APOBEC3B* copy number is thus also not a predictive biomarker for the type of response to chemotherapy.

In a previous study, we had analyzed *APOBEC3B* mRNA expression in 1,491 breast cancer patients and found that high *APOBEC3B* expression had prognostic value and was associated with poor outcome in untreated LNN patients with ER-positive breast cancer [15]. However, we did not find any association between *APOBEC3B* copy numbers and clinical outcome in the current study. As both *APOBEC3B* expression and *APOBEC3B* copy number have been associated with a hypermutator phenotype [13,14,24], we attempted to clarify this discrepancy by evaluating the correlation between *APOBEC3B* copy number and *APOBEC3B* mRNA expression. Out of the 1,756 patients for whom we performed *APOBEC3B* copy number analysis in the current study, we had *APOBEC3B* mRNA expression data available for 1,132 patients. Interestingly, although a correlation among *APOBEC3B* copy number and mRNA expression was observed, the correlation coefficient was low (spearman’s rho = 0.26, *P* = 2.2*10^−16^). This suggests that other mechanisms exist that affect *APOBEC3B* mRNA expression besides *APOBEC3B* copy number.

## Discussion

*APOBEC3B* mRNA expression is upregulated in multiple tumor types and this has been shown to correlate with an increased mutational load, particularly an increase in C>T transversions [13,14]. In line with these findings, increased expression of *APOBEC3B* mRNA was associated with a poor prognosis in ER-positive breast cancer [15]. At the same time, the 29.5 kb deletion polymorphism of *APOBEC3B* was found to be associated with the increased breast cancer risk in different populations [19–21], although these findings were not confirmed in a Swedish study [22]. Similar to *APOBEC3B* overexpression, the *APOBEC3B* deletion has been shown to correlate with an increased mutational load [24]. These findings may seem paradoxical, as loss of *APOBEC3B* should decrease the mutational load. However, the *APOBEC3B* deletion polymorphism not just deletes *APOBEC3B*. It also generates a novel fusion transcript (*i*.*e*. *APOBEC3A* under the control of the 3’UTR of *APOBEC3B*) [18]. Consequently, *APOBEC3A* mRNA was shown to become more stable, resulting in higher levels of APOBEC3A and, since APOBEC3A is a more efficient hypermutator than APOBEC3B, more severe DNA damage [23]. Thus, although the molecular mechanisms behind overexpression of *APOBEC3B* and the *APOBEC3B* deletion polymorphism are very different, they both result in a hypermutator phenotype. The hypothesis that the *APOBEC3B* deletion polymorphism thus may also be associated with clinical outcome is therefore plausible.

In a study by Gohler *et al*., the *APOBEC3B* deletion polymorphism was however, not associated with breast cancer specific survival in 782 breast cancer cases [22]. Moreover, Cescon *et al*. showed that the deletion was not associated with recurrence after treatment for early breast cancer in METABRIC [16]. Because both studies also included patients that were treated, no distinction could be made between pure prognosis and therapy response. In the current study, we examined separately the prognostic and predictive value of *APOBEC3B* copy number in a total of 1,756 breast cancer patients. No association between *APOBEC3B* copy numbers and the length of MFS was found among 1,076 LNN patients who had not received adjuvant systemic treatment (Fig 2B, Table 2). In addition, an association with the length of MFS was also not observed among ER-positive or ER-negative breast cancer patients. These results imply that *APOBEC3B* copy number is not a prognostic biomarker for breast cancer. The association between *APOBEC3B* copy number and the response to treatment in breast cancer patients that received either first-line tamoxifen or chemotherapy for recurrent disease was also evaluated. However, we found no association between the type of response for either tamoxifen or chemotherapy and *APOBEC3B* copy number. Thus, besides not having any prognostic value, *APOBEC3B* copy numbers also do not appear to have a predictive value for breast cancer patients.

**Fig 3:**
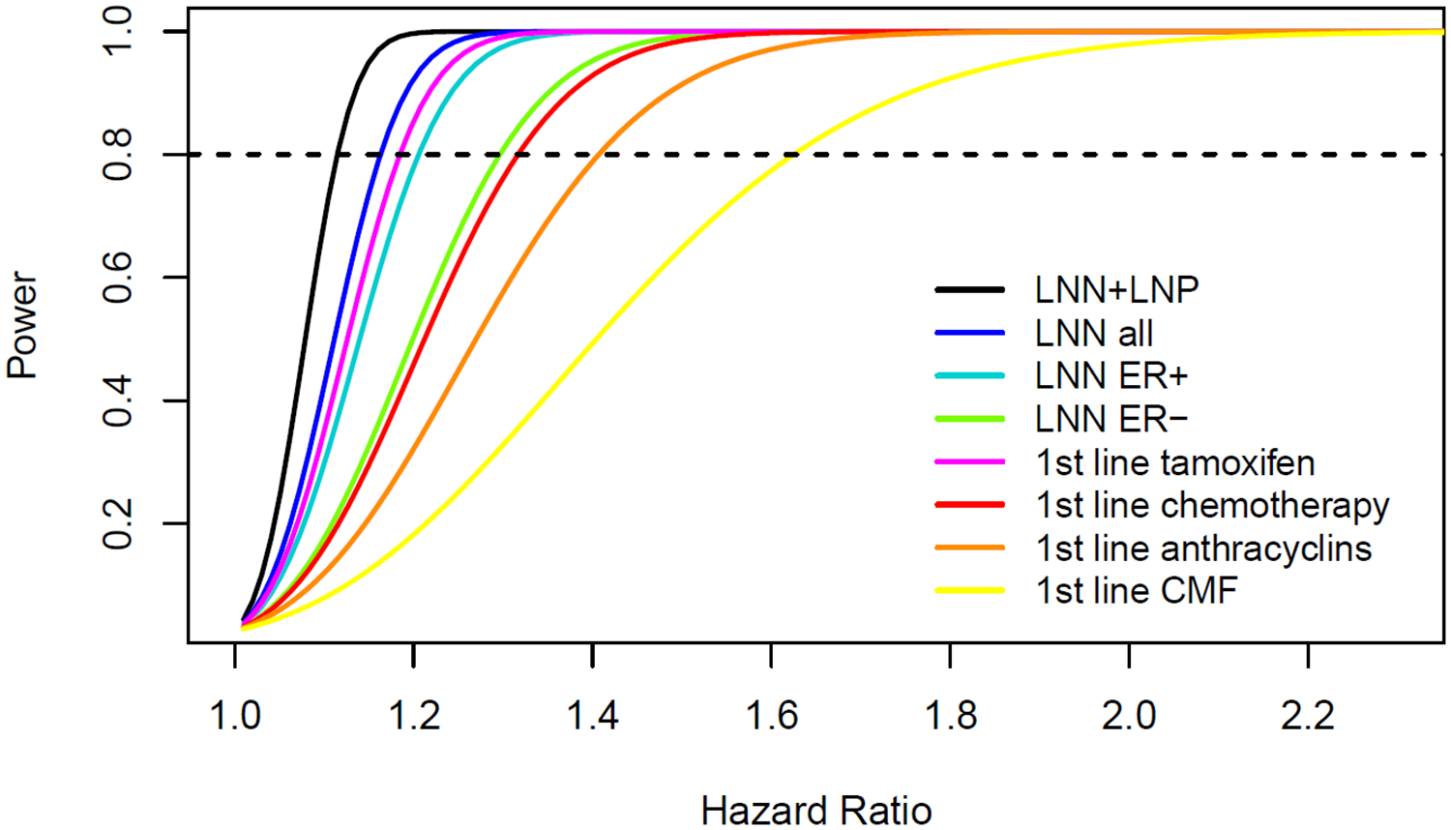
Power as a function of the hazard ratio for *APOBEC3B* copy number in each of the analyzed subgroups. The dashed horizontal line crosses the curve of each subgroup at the minimal hazard ratio for which we had 80% power in our *APOBEC3B* copy number analyses.

The copy number analyses in the adjuvant setting, except for the ER-negative LNN analysis, had sufficient power to conclude that there is no or only a marginal prognostic effect of *APOBEC3B* copy numbers in breast cancer patients (Fig 3). For the copy number analysis in the advanced setting involving patients treated with first-line tamoxifen, we could draw the same conclusion (Fig 3). However, for the copy number analysis involving patients treated with first-line chemotherapy, especially in the subgroup analyses by type of chemotherapy, we cannot exclude a modest effect of *APOBEC3B* copy numbers on the length of PFS (Fig 3). Therefore, replication of the results we observed in the advanced chemotherapy setting in a larger sample size or population with a higher prevalence of the *APOBEC3B* deletion polymorphism would be needed.

Another note is that the assay we used to determine *APOBEC3B* copy numbers does not discriminate between germline and tumor-specific (*i*.*e*. somatic) *APOBEC3B* deletion. This has no consequence for the accuracy of the germline copy number determination except for patients who carry a one-copy deletion and have strong amplification of the remaining *APOBEC3B* locus. Patients who do not carry the germline deletion and have either a tumor-specific homozygous deletion or a heterozygous deletion of *APOBEC3B* in combination with a high tumor cell percentage may also be misclassified. In this respect, the observed amplification of the *APOBEC3B* allele is rather a somatic event than the result of an alteration in the germline. To date, there has been no report of the *APOBEC3B* locus being amplified in the germline. Interestingly, in a publically available SNP array data set of 344 breast cancers (accession number EGAS00001001178 [33]), *APOBEC3A* copy number status was equal to *APOBEC3B* copy number status in all tumors, suggesting that *APOBEC3A* is always amplified or deleted simultaneously with *APOBEC3B* during breast tumorigenesis.

In our Kaplan-Meier survival analysis we grouped patients with one-copy deletion and two-copy deletions together to provide a visualization of the estimated survival curves of patients with a deleted, balanced or amplified *APOBEC3B* gene. Unfortunately, the population frequency of the 29.5 kb deletion polymorphism of *APOBEC3B* was too low in this Dutch cohort to analyze patients who carry a two-copy deletion separately. To illustrate this with a power calculation: we would need a sample size of 4,024 or 11,759 LNN patients to ensure a minimally detectable hazard ratio of 2 or 1.5, respectively, using a power of 80% and an alpha of 0.05. For this reason, evaluation of the clinical value of the two-copy deletion should preferably be done in breast cancer patients from the Asian or American population (*i*.*e*. population frequency of 36.9% and 57.7%, respectively)[18].

The observation that the *APOBEC3B* deletion polymorphism does not seem to have any impact on the clinical outcome for breast cancer patients, whereas increased expression of *APOBEC3B* mRNA does, strengthens the evidence that there are two different molecular mechanisms in place. Moreover, elevated levels of *APOBEC3B* mRNA have been shown to associate with cellular proliferation, whereas the *APOBEC3B* deletion polymorphism associated with activation of immune-related genes [16]. Interestingly, increased lymphocytic infiltration has been associated with a favorable outcome in some, but not all subtypes of breast cancer [34,35]. This is in contrast to what has been observed for increased *APOBEC3B* mRNA expression, which associated with increased proliferation and poor outcome [15].

Although we observed a correlation between *APOBEC3B* copy number and mRNA expression, this correlation was rather weak. As a consequence, the prognostic effect of increased levels of *APOBEC3B* mRNA is not necessarily caused by increased *APOBEC3B* copy numbers. Other mechanisms thus exist that elevate *APOBEC3B* mRNA levels. Recently, it was shown that APOBEC3B interacts with ER to bind to ER binding sites, where it generates C to U transitions. Furthermore, the presence of APOBEC3B was necessary for histone modification, but also in order to recruit chromatin remodeling factors to ER binding sites [36]. This may very well explain why elevated levels of *APOBEC3B* mRNA are only prognostic in ER-positive breast cancer patients. Although the *APOBEC3B* deletion polymorphism does not have any prognostic or predictive value, it does appear to contribute to breast tumorigenesis by conferring an increased risk to develop breast cancer. However, still little is known regarding the role of APOBEC3A in breast cancer. Therefore, more studies should be done in order to investigate the precise effect of the *APOBEC3B* deletion polymorphism and *APOBEC3A-B* hybrid transcript resulting from this deletion. This will provide more insight into APOBEC-mediated hypermutation.

## Supporting Information

S1 Fig Histogram of the ΔCt values for all 1,756 measured DNA samples and cell line OCUB-F. (PDF)

S1 Table Clinical data of all 1,756 breast cancer patients included in the study. (XLSX)
