# Supplementary material for: The 29.5 kb APOBEC3B Deletion Polymorphism Is Not Associated with Clinical Outcome of Breast Cancer
Source: PLoS One. 2016 Aug 23;11(8):e0161731. doi: 10.1371/journal.pone.0161731 (PMC4995039; doi:10.1371/journal.pone.0161731)
Supplement: S1 Fig — (PDF) [file pone.0161731.s001.pdf]

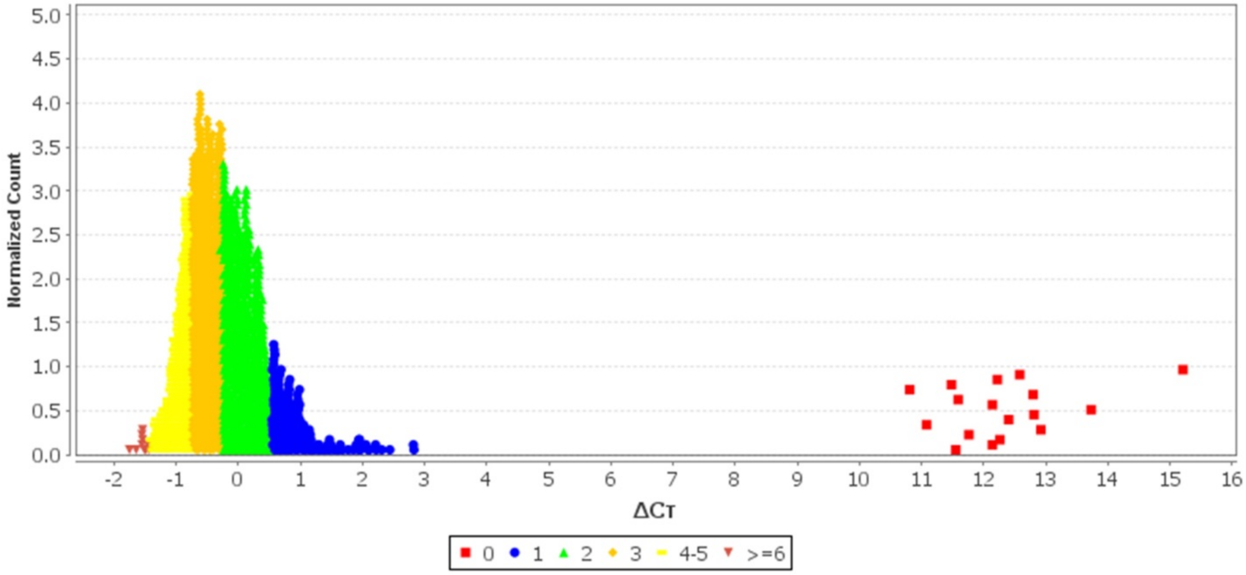

S1 Fig. Histogram of the ΔCt values for all 1,756 measured DNA samples and cell line OCUB-F. The ΔCt value was calculated by subtracting the Ct value for the target gene (*i.e. APOBEC3B*) from the Ct value of the reference gene (*i.e. RNase P*). Then ΔCt values were converted to calculated copy number values by the CopyCaller software and grouped into 6 predicted copy number categories. Samples with ΔCt values ≥ 3.00 or calculated copy number values ≤ 0.20 were grouped into category "0 copies" (red). Samples with ΔCt values ≥ 0.55 and < 3.00 or calculated copy number values > 0.20 and ≤ 1.41 were grouped into category "1 copy" (blue). Samples with ΔCt values ≥ -0.29 and < 0.55 or calculated copy number values > 1.41 and ≤ 2.52 were grouped into category "2 copies" (green). Samples with ΔCt values ≥ -0.74 and < -0.29 or calculated copy number values > 2.52 and ≤ 3.44 were grouped into category "3 copies" (orange). Samples with ΔCt values ≥ -1.40 and < -0.74 or calculated copy number values > 3.44 and ≤ 5.44 were grouped into category "4-5 copies" (yellow). Samples with ΔCt values < -1.40 or calculated copy number values > 5.44 were grouped into category "≥ 6 copies" (brown). For analyses purposes, we further reduced the six predicted copy number categories to four categories: "two-copy deletion", "one-copy deletion", "balanced" and "amplified". This was achieved by combining the "2 copies" and "3 copies" categories into the "balanced" category and by combining the "4-5 copies" and "≥ 6 copies" categories into the "amplified" category.
